# Genome‐wide identification and functional profile analysis of long non‐coding RNAs in *Avicennia marina*

**DOI:** 10.1002/tpg2.20450

**Published:** 2024-04-11

**Authors:** Lingling Wang, Zixin Yuan, Jingyi Wang, Yali Guan

**Affiliations:** ^1^ Ministry of Education Key Laboratory for Ecology of Tropical Islands, Key Laboratory of Tropical Animal and Plant Ecology of Hainan Province, College of Life Sciences Hainan Normal University Haikou China; ^2^ Hainan Observation and Research Station of Dongzhaigang Mangrove Wetland Ecosystem Haikou China

## Abstract

*Avicennia marina*, known for its remarkable adaptability to the challenging coastal environment, including high salinity, tide, and anaerobic soils, holds pivotal functions in safeguarding the coastal ecosystem. Long non‐coding RNAs (lncRNAs) have emerged as significant players in various natural processes of plants such as development. However, lncRNAs in *A. marina* remain largely unknown and uncharacterized. Here, we employed the transcriptome datasets from multiple tissues, such as root, leaf, and seed, to detect and characterize the lncRNAs of *A. marina*. Analyzing synthetically, we finally identified 6333 lncRNAs in the *A. marina*. These lncRNAs exhibited distinct features compared to messenger RNAs, including larger exons, lower guanine‐cytosine contents, lower expression levels, and higher tissue specificities. Moreover, we identified thousands of tissue‐specific lncRNAs across the examined tissues and further found that these tissue‐specific lncRNAs were significantly enriched in biological processes related to the major functions of their corresponding tissues. For instance, leaf‐specific lncRNAs showed prominent enrichment in photosynthesis, oxidation–reduction processes, and light harvesting. By providing a comprehensive dataset and functional annotations for *A. marina* lncRNAs, this study offers a valuable overview of lncRNAs in *A. marina* and lays the fundamental foundation for further functional exploring of them.

## Core Ideas

- *Avicennia marina* thrives in a harsh ecological environment and plays an important role in coastal ecosystem safeguarding. However, molecular mechanisms are still in its infancy.
- We present an overview of lncRNAs of multiple tissues from *Avicennia marina* based on RNA‐seq data analysis.
- The features of these lncRNAs were analyzed, and their biological functions were annotated and enriched.
- The functions of identified tissue‐specific lncRNAs help elucidate the development mechanisms of *Avicennia marina*.
- Our research will lay theoretical foundations for further functional studies of *Avicennia marina* long non‐coding RNAs.

**Abbreviations**
CPC2: coding potential calculator 2
GC: guanine and cytosine
lncRNAs: long non‐coding RNAs
LRK: leucine‐rich repeat receptor kinase
ncRNAs: non‐coding RNAs
ORF: open reading frame
*PID*
: *PINOID*

## 1 INTRODUCTION

More than 90% of eukaryotic genomes are transcribed (Ariel et al., 2015). However, only approximately 2% of these transcripts get translated, the majority are non‐coding RNAs (ncRNAs) possessing little to no discernable protein‐coding ability (Chekanova et al., 2007; Kapranov et al., 2007; Wilusz et al., 2009). ncRNAs can be classified into different types according to the nucleotide (nt) lengths (Yu et al., 2019). Long non‐coding RNAs (lncRNAs) are RNA transcripts longer than 200 nt, without coding capacity, and constitute one of the most extensive categories of ncRNAs (Charles Richard & Eichhorn, 2018; Derrien et al., 2012; Quinn & Chang, 2016). Mounting evidence has substantiated the significance of lncRNAs as vital regulators participating in a wide array of biological processes as development and environmental responses (Ariel et al., 2015; Wierzbicki, 2012).

Within animal kingdom, the functions and regulation mechanisms of lncRNAs have been well studied (Anastasiadou et al., 2018; Batista & Chang, 2013; Bond et al., 2009; Fatica & Bozzoni, 2014; Zhou et al., 2020), while the genome‐wide identification and functional explorations of plant lncRNAs are far behind and less comprehensive. In recent years, benefiting from advanced sequencing technology and bioinformatics analysis, numerous plant lncRNAs have been identified and reported. Following, the biological functions of several lncRNAs have been clarified in certain plant species. In *Arabidopsis*, 200 transcriptome datasets were used to identify lncRNAs; as a result, more than 36,000 candidates, including natural antisense transcripts (>30,000) and long intergenic ncRNAs (lincRNAs) (>6000), were found (Jin et al., 2013; Wang, Chung, et al., 2014). In addition, *HIDDEN TREASURE 1*, a 236 nt length lncRNA, can promote *Arabidopsis* photomorphogenesis by regulating the expression of *PHYTOCHROME INTERACTING FACTOR 3* and *PROTOCHLOROPHYLLIDE OXIDOREDUCTASES* (Wang, Fan, et al., 2014; Wang, Li, et al., 2018). The *AUXIN REGULATED PROMOTER LOOP RNA*/npc34 lncRNA, which is located upstream of *PINOID* (*PID*, coding an auxin signaling kinase) gene, positively regulates the expression of *PID* and *ROOT HAIR DEFECTIVE 6* and then has a significant effect on root development (Moison et al., 2021). Besides, *Arabidopsis* lncRNAs are reported to be involved in regulating flowering (Tian Y et al., 2019) and biotic and abiotic stress responses (Liu et al., 2019; Qin et al., 2017). In maize, 1535 drought‐responsive lncRNAs are identified under arid stress (Pang et al., 2019). Furthermore, over‐expression of LRK (leucine‐rich repeat receptor kinase) antisense intergenic RNA (*LAIR*) enhances grain yield by promoting the expression of LRK neighboring genes in rice (Wang, Luo et al., 2018). To date, lncRNAs have been identified and studied not only in *Arabidopsis*, maize, and rice (Huang et al., 2021) but also in other non‐model plant species, such as rubber (Li et al., 2021; Liu et al., 2022; Wang et al., 2022; Yin et al., 2019).

*Avicennia marina* (Forssk.) Vierh. (Acanthaceae) is a mangrove tree with a broad distribution, which is known as the guardian of the coast and plays important ecological as well as environmental roles in protecting the water quality, maintaining coast stability, mitigating climate change, conserving biodiversity, etc. (Kuenzer et al., 2011). As the main species of the coastal mangrove ecosystem, *A. marina* can survive in extreme ecological environments, such as external salt concentration and heavy metal toxicity (Zeinali et al., 2017). However, the relevant molecular mechanisms implicated in regulating the development, growth, and environmental adaptions of *A. marina* under different conditions were still largely unknown. Most of the previous studies are mainly focused on physiology and biochemistry studies or the functional role of coding genes (Kavitha et al., 2008; Natarajan et al., 2021). In recent years, although an accumulating number of research indicates that lncRNAs are significant regulators involved in plant development and stress responses (Moison et al., 2021; Tian et al., 2023), the identification, characterization, and biological function exploration of lncRNAs in *A. marina* was still unreported.

To overview the quantity, types, and features, as well as pursue further biological function information about the lncRNAs of *A. marina*, we conducted comprehensive identification, characterization, and annotation of them with transcriptome datasets from root, pneumatophore, stem, leaf, flower, seed, etc., tissues. These results will provide valuable insights and clues to understand the functions of *A. marina* lncRNAs and lay theoretical foundations for further functional studies of *A. marina* lncRNAs.

## 2 MATERIALS AND METHODS

### 2.1 Transcriptome assembly of long and short reads

**FIGURE 1:**
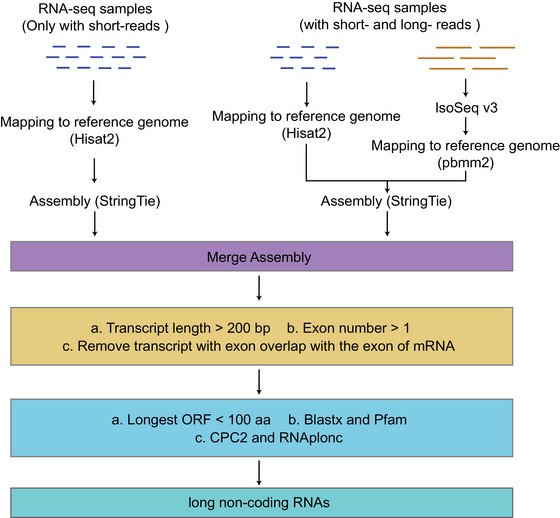
The pipeline to identify the long non‐coding RNAs (lncRNAs) in *Avicennia marina*. This pipeline demonstrates the process from transcriptome assemble to the lncRNA identification. The short blue line represents the short reads RNA‐seq data, while the long sandybrown line indicates the long reads RNA‐seq data. ORF, open reading frame; mRNA, messenger RNAs; CPC2, coding potential calculator 2.

The RNA‐seq datasets were obtained from the sequence read archive (SRA) database (https://www.ncbi.nlm.nih.gov/sra) of NCBI (National Center for Biotechnology Information) (Table S1). The reference genome of *A. marina* was downloaded from NCBI (https://www.ncbi.nlm.nih.gov) (Natarajan et al., 2021). The FastQC (v0.11.9) was used to check the RNA sequencing quality (Andrews, 2010) and AdapterRemoval (v2.3.1) was applied to remove remnant adapter sequences from the sequencing reads (Schubert et al., 2016). For the samples only with short‐read RNA sequencing data, we mapped the reads to the genome sequence of *A. marina* using hisat2 (v2.2.1) (Figure 1) (Kim et al., 2019). For the PacBio long reads, we followed the pipeline in Isoseq3 (https://github.com/PacificBiosciences/IsoSeq) to generate the potential isoforms and utilized the pbmm2 to map them to the reference genome. Stringtie (v2.2.1) was used to assemble the reads to potential transcripts following the read mapping for each sample (Figure 1) (Kovaka et al., 2019). For the samples with both short and long reads, we use both of them for the transcriptome assembly. We merged the transcripts from different samples into a non‐redundant set of transcripts and filtered them without strand information.

### 2.2 Identification of lncRNAs

To provide a comprehensive dataset for lncRNAs of *A. marina*, we identified the lncRNAs from RNA‐sequencing data generated from multiple tissues, including root, leaf, and seed. First, the sequences were filtered with the following criteria: transcripts with length >200 bp and exon number >1 were retained, and the transcripts with exon overlapped with messenger RNAs (mRNAs) were removed (Li et al., 2014; Zhang et al., 2014). Next, we screened the transcripts based on their coding potential. The coding potential was assessed by (a) the longest open reading frame (ORF) length, (b) sequence similarity or domain with the known proteins, and (c) coding score. The longest ORF was calculated by the TransDecoder software with the parameter of ‐m 30 & ‐S (https://github.com/TransDecoder/TransDecoder). The transcripts that encode less than 100 aa (amino acid) were preserved and then used for sequence alignments (Blastx, *e*‐value = 1e‐3) and domain scan (pfamscan, ‐e_seq = 1e‐3 and ‐e_dom = 1e‐3) to remove the transcripts containing significant homology with the known protein sequences or Pfam domains (Camacho et al., 2009; Mistry et al., 2021). To further recognize the non‐coding transcripts, we utilized coding potential calculator 2 (CPC2) and RNAplonc (v1.1) to calculate their coding scores, and eliminated the coding transcripts with default criteria (Kang et al., 2017; Negri et al., 2019). The remaining transcripts were recognized as the lncRNAs of *A. marina*.

**Core Ideas**

- *Avicennia marina* thrives in a harsh ecological environment and plays an important role in coastal ecosystem safeguarding. However, molecular mechanisms are still in its infancy.
- We present an overview of lncRNAs of multiple tissues from *Avicennia marina* based on RNA‐seq data analysis.
- The features of these lncRNAs were analyzed, and their biological functions were annotated and enriched.
- The functions of identified tissue‐specific lncRNAs help elucidate the development mechanisms of *Avicennia marina*.
- Our research will lay theoretical foundations for further functional studies of *Avicennia marina* long non‐coding RNAs.

### 2.3 Classification distribution of *A. marina* lncRNAs

We employed the FEELnc to sort the identified lncRNAs into different categories, including intergenic lncRNAs, antisense lncRNAs, and intronic lncRNAs, and other lncRNAs based on the localization and transcriptional direction of proximal mRNAs (Wucher et al., 2017).

### 2.4 Chromosome distribution of lncRNAs in *A. marina*

To examine the chromosomal assignment of lncRNAs, we first calculated the number and density of lncRNAs for each chromosome. The lncRNAs density is calculated with the following equation:

$$\mathrm{lncRNAs}\;\mathrm{density}=\frac{\mathrm{Total}\;\mathrm{lncRNA}\;\mathrm{of}\;\mathrm{ChrN}}{\mathrm{Length}\;\mathrm{of}\;\mathrm{ChrN}}\times 10^{6}$$

where ChrN indicates the chromosome N.

Subsequently, we split each chromosome into 200‐kb‐width bins from the start site to the end with a 200 kb step, then counted the number of lncRNAs and mRNA in each bin, separately.

### 2.5 Sequence characteristics of *A. marina* lncRNAs

We inspected and contrasted the sequence characteristics, including exon size, exon number, transcript length, and guanine and cytosine (GC) content, between lncRNAs and mRNAs. We used R software (v4.0.2, https://cran.r‐project.org) and R package (GenomicFeatures v1.40.1, https://bioconductor.org/packages/release/bioc/html/GenomicFeatures.html) to compute the exon size, intron size, exon number, and transcript length (Lawrence et al., 2013). Meanwhile, the LncFinder (v1.1.5, a R package) was utilized to calculate GC contents for the lncRNAs and mRNAs (Han et al., 2019).

### 2.6 Expression profile

In order to overview and compare the expression levels of *A. marina* mRNAs and lncRNAs, we used the salmon (v1.4.0) to calculate the raw read counts for each transcript (Patro et al., 2017). The raw read counts were normalized by the trimmed mean of the M‐values (TMM) approach and converted to fragments per kilobase of sequence per million mapped reads with edgeR (v3.30.3) (Robinson et al., 2010).

### 2.7 Tissue‐specificity analysis

We adopted the expression data from five tissues (root, leaf, stem, flower, and seed) to investigate the tissue‐specificity of lncRNAs and mRNAs. The fractional expression for each RNA (lncRNA or mRNA) in a given tissue was the proportion of its expression against the total expressions of this RNA across all five tested tissues (Ding et al., 2018). The maximum fractional expression for each lncRNA is defined as its tissue‐specific score across the examined tissues. The criterion of tissue‐specific score >0.6 is employed to identify the tissue‐specific lncRNAs (Wang et al., 2022).

### 2.8 Functional analysis for lncRNAs

Generally, lncRNA is tightly correlated with its potential targets in the expression level, the function of lncRNA can be predicted by its co‐expression mRNAs. Thus, we conducted the functional analysis for the *A. marina* lncRNAs depending on the functional enrichment of their co‐expression mRNAs. We performed the Pearson correlation analysis between lncRNAs and mRNAs and employed the criteria of Pearson correlation >0.5 and adjusted *p*‐value < 0.05 to find the co‐expression mRNAs for each lncRNA. The gene ontology (biological process category) for the co‐expression mRNAs of lncRNAs was annotated by the software interproscan (v5.48‐83.0) with the parameter of ‐pa ‐goterms (Jones et al., 2014). We conducted the enrichment analysis for the co‐expression mRNAs of each lncRNA to carry out function annotations using hypergeometric test (Kolberg et al., 2020). The *p*‐value adjusted by Benjamini and Hochberg and a cutoff of adjusted *p*‐value < 0.1 was used to determine the enrichment significance (Benjamini & Hochberg, 1995). Only the significantly enriched gene ontologies were used to annotate the function of lncRNAs. The functional enrichment analysis for the tissue‐specific lncRNAs is also operated by the hypergeometric test with the same criteria.

## 3 RESULTS

### 3.1 Identification of lncRNAs in *A. marina*

To provide a comprehensive profile of lncRNAs in *A. marina*, we employed the RNA‐seq datasets that included both long reads and short reads sequencing data from multiple tissues to identify the lncRNAs (Figure 1 and Table S1). The reads were mapped to reference genome of *A. marina* and subsequently assembled into transcripts. Though merging the transcripts from different samples, we obtained a total of 123,183 transcripts.

We identified lncRNAs in *A. marina* through a two‐step process. Initially, we filtered the transcripts based on their lengths, exon numbers and exon overlapped with mRNA, and obtained 11,443 residual transcripts. Subsequently, we conducted a screening process based on coding potential, which was assessed by ORF length, Blastx, CPC2, etc. As a result, 6333 lncRNAs were finally identified in *A. marina*.

### 3.2 Distribution and category of *A. marina* lncRNAs

**FIGURE 2:**
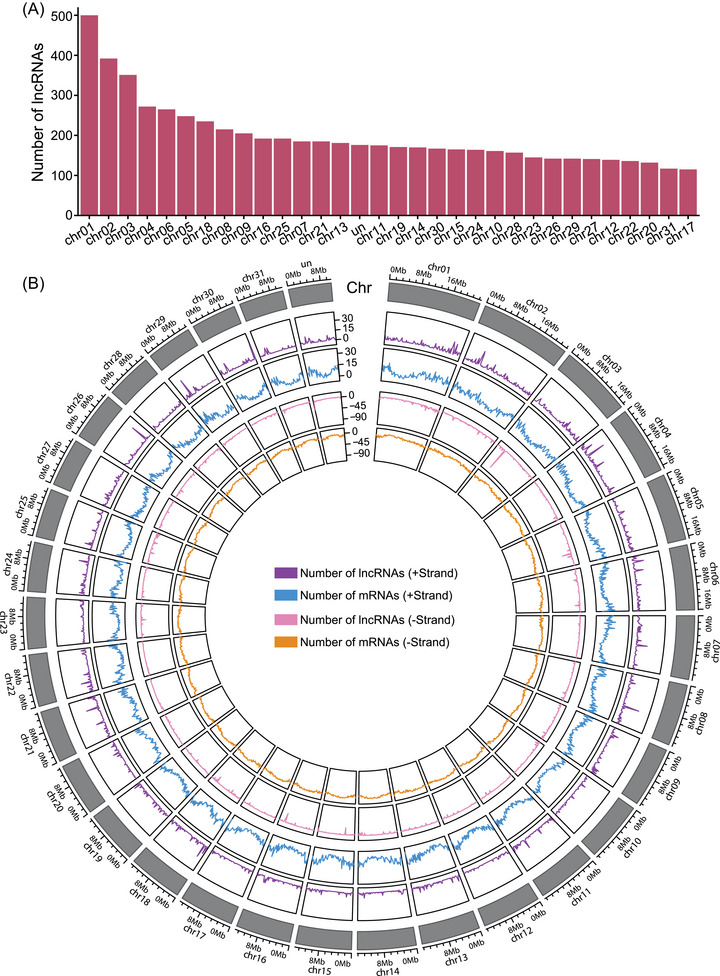
Long non‐coding RNAs' (lncRNAs) distribution across the chromosomes. (A) Number of lncRNAs across the chromosomes. (B) The distribution of lncRNAs and messenger RNAs (mRNAs) on each chromosome. The out layer represents the chromosomes. The purple, blue, pink, and orange colors indicate the lncRNAs on + strand, mRNAs on + strand, lncRNA on − strand, and mRNA on − strand, respectively.

At the chromosome level, we observed that chromosomes 1, 2, and 3 contained more than 300 lncRNAs each (Figure 2A). Notably, both lncRNAs and mRNAs exhibited a preferred localization on these particular chromosomes (Figure 2A and Figure S1A). The number of lncRNAs and mRNAs on each chromosome may be influenced by chromosome's length. To account for this potential bias, we normalized the lncRNA and mRNA counts by scaling the chromosome length to 10^6^. Notably, even after this normalization, chromosomes 1, 2, and 3 were still ranking the top chromosomes in terms of lncRNA and mRNA distribution (Figure S1B,C). To further examine the detailed distribution of *A. marina* lncRNAs on each chromosome, we calculated the number of lncRNAs within 20 kb sliding window across each chromosome. Our analysis revealed that the distribution of lncRNAs on the same chromosome differed to some extent from that of mRNAs (Figure 2B). For instance, mRNAs exhibited relatively less distributions in the middle region of the chromosomes, whereas lncRNAs did not (Figure 2B). Furthermore, considering the genomic location relationship between lncRNAs and mRNAs, we categorized the lncRNAs into different categories, including intergenic, antisense, and intronic lncRNAs. Notably, more than 4000 lncRNAs fell into the category of intergenic lncRNAs (Figure S1D). Approximately 30% and 3% of the lncRNAs were identified as antisense and intronic lncRNAs, respectively (Figure S1D).

### 3.3 Distinct features of lncRNAs in comparison with mRNAs

**FIGURE 3:**
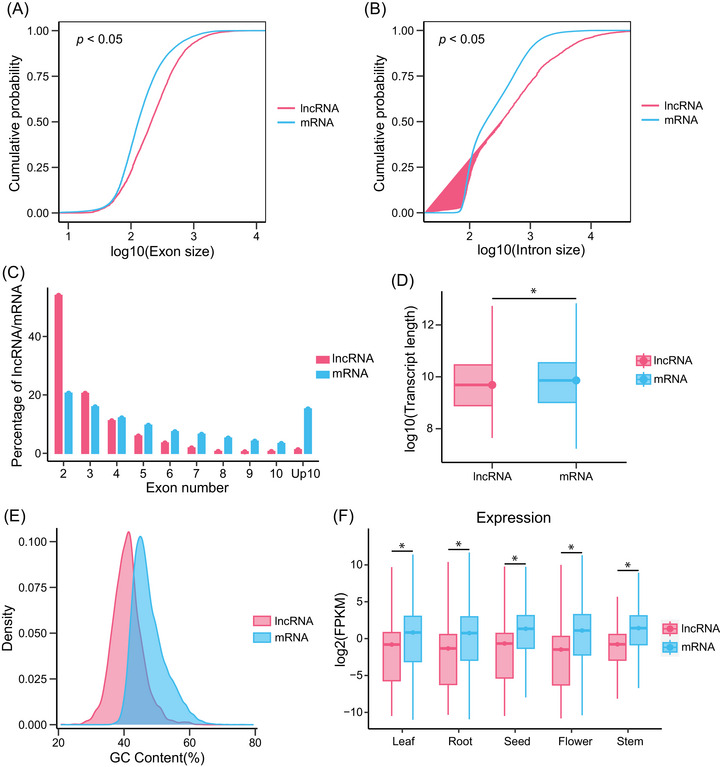
Characteristics of long non‐coding RNAs (lncRNAs) in *Avicennia marina*. The cumulative probability of (A) exon size and (B) intron size of lncRNAs and messenger RNAs (mRNAs). *p*‐Value was calculated by Kolmogorov–Smirnov test. (C) Percentage of lncRNAs and mRNAs with different exon numbers. (D) The transcript length of lnRNAs and mRNAs. The statistical significance was performed by the Mann–Whitney test (*p < 0.05). (E) Density distribution of guanine and cytosine (GC) content (%) in lncRNAs and mRNAs. (F) The expression level of lncRNAs and mRNAs; *Y* axis indicates the expression level (log2(FPKM), where FPKM is fragments per kilobase of sequence per million mapped reads). The statistical significance was conducted by the Mann–Whitney test (*p < 0.05).

To investigate whether lncRNAs possess particular features, we examined and compared exon size, exon number, transcript length, and GC content between lncRNAs and mRNAs. The results indicated that lncRNAs exhibit distinct characteristics, such as larger exon sizes and longer introns than mRNAs (Figure 3A,B). These features are consistent with the characteristics of lncRNAs in other plants, such as *Hevea brasiliensis* and *Solanum lycopersicum* (Wang et al., 2022, Wang, Zhao, et al., 2018). Additionally, we observed that lncRNAs in *A. marina* tend to contain fewer exons than mRNAs (Figure 3C). As approximately 15% mRNAs contain >10 exons, only ∼3% lncRNAs possess over 10 exons, suggesting that lncRNAs in *A. marina* commonly have relatively fewer exons compared to mRNAs. Furthermore, we found that lncRNAs in *A. marina* have shorter sequence lengths than mRNAs (Figure 3D), which is consistent with the features of lncRNAs in soybean and *Camellia sinensis* (Lin et al., 2020; Wan et al., 2020).

In general, lncRNAs are known to possess lower GC content than mRNAs (Haerty & Ponting, 2015). Consistently, we found that lncRNAs in *A. marina* also exhibit lower GC content compared to mRNAs (Figure 3E). It suggests that the lower GC content in lncRNAs may potentially affect the coding potential of their sequences. Moreover, we observed that lncRNAs exhibit lower expression levels than mRNAs across the examined tissues (Figure 3F), suggesting that GC content may also associate with the potential of DNA transcription in *A. marina*.

### 3.4 Tissue specificity of lncRNAs and mRNAs

To assess the tissue specificities of both lncRNAs and mRNAs, we utilized expression data from various tissues to calculate the tissue‐specific scores for them.

Using a criterion of tissue‐specific score >0.6, we identified more than a thousand of tissue‐specific lncRNAs from the examined tissues (Figure S2A). Most of these tissue‐specific lncRNAs were primarily identified in the leaf and seed tissues, indicating their important functions in seed and leaf development, growth, as well as physiology (Figure S2A,B). Additionally, around 200 lncRNAs were respectively identified as tissue‐specific in flower and root tissues, and only about 100 members were found to be the stem tissue‐specific lncRNAs (Figure S2A).

**FIGURE 4:**
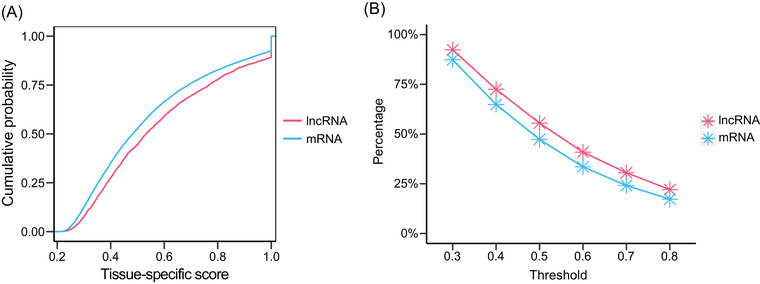
Tissue specificity of the long non‐coding RNAs (lncRNAs) and messenger RNAs (mRNAs). (A) Cumulative probability of tissue‐specific score of lncRNAs and mRNAs. (B) Percentage of tissue‐specific lncRNAs and mRNAs under varying thresholds of the tissue‐specific score.

Moreover, as shown in Figure 4A, we found that lncRNAs exhibited significantly higher tissue‐specific scores compared with mRNAs. Meanwhile, regardless of the threshold used to define tissue‐specific RNAs, we consistently observed a relatively higher percentage of tissue‐specific lncRNAs than mRNAs (Figure 4B). This consistent tendency demonstrated that lncRNAs possess higher tissue specificities in *A. marina*, indicating their potential roles in regulating the development, growth, and biological function processes of the particular tissues.

### 3.5 Tissue‐specific lncRNAs exhibit tissue‐specific functional relevance

Although the aforementioned analysis has successfully identified around a thousand of tissue‐specific lncRNAs in *A. marina*, their functional roles remain unclear. To unravel the potential functions of tissue‐specific lncRNAs, we employed functional annotation of co‐expression mRNAs to predict their biological functions.

**FIGURE 5:**
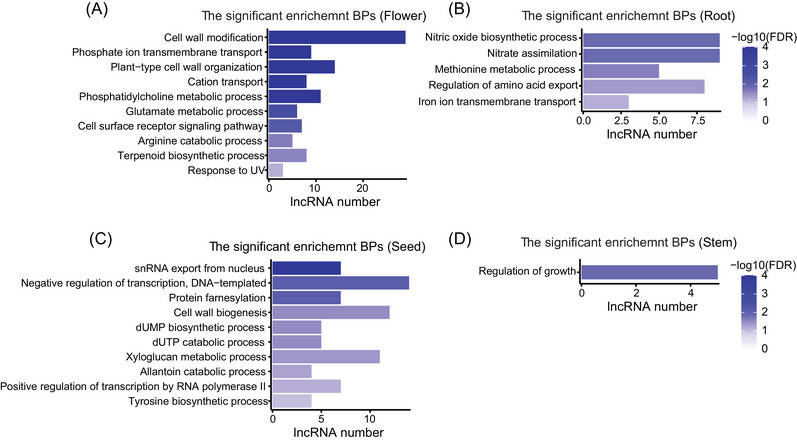
Functional enrichment for the tissue‐specific long non‐coding RNAs (lncRNAs). The significant enrichment BPs (biological processes) for tissue‐specific lncRNAs in (A) flower, (B) root, (C) seed, and (D) stem. FDR, false discovery rate.

The functional analysis revealed that flower‐specific lncRNAs are significantly enriched in the biological processes related to the cell wall modification and organization (Figure 5A and Table S2). While the root tissue‐specific lncRNAs were predominantly enriched in the nitric oxide biosynthetic process (Figure 5B and Table S3). In the case of seed‐specific lncRNAs, they were found to be associated with transcriptional regulation and cell wall biogenesis processes (Figure 5C and Table S4), both of which are connected to the preparation for the seed development. Differently, the stem‐specific lncRNAs were enriched in the biological process of growth regulation (Figure 5D and Table S5), suggesting their potential roles in regulating the developmental and living processes of stems.

**FIGURE 6:**
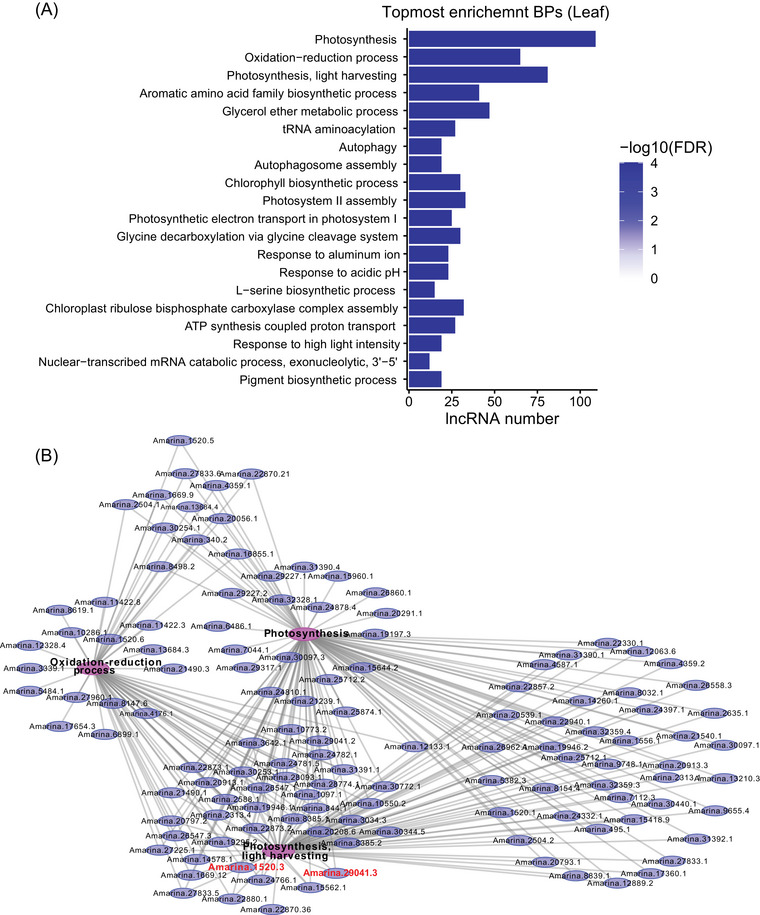
Functional enrichment for the leaf‐specific long non‐coding RNAs (lncRNAs). (A) The topmost enrichment BPs (biological processes) of the leaf‐specific lncRNAs. (B) The network of leaf‐specific lncRNAs related to the three topmost enrichment BPs. The rose red nodes indicate the BPs, while blue nodes represent the lncRNAs.

In comparison to other tissues, leaf‐specific lncRNAs are enriched in a greater number of biological processes (Figure 6A and Table S6). Remarkably, the topmost enriched pathways for leaf‐specific lncRNAs include photosynthesis, oxidation–reduction processes, and light harvesting (Figure 6A), which are highly relevant to the main function of leaf tissues. Notably, dozens of leaf‐specific lncRNAs, including *Amarina.29041.3* and *Amarina.1520.3*, are involved in these biological processes (Figure 6B), emphasizing the contribution of leaf‐specific lncRNAs to the primary function of leaves. Furthermore, we discovered that leaf‐specific lncRNAs, such as *Amarina.29041.3* and *Amarina.1520.3*, not only regulate the major functions of leaves but also participate in other biological processes, such as translation, defense responses, and response to gibberellin (Figure S3 and Table S7), indicating their crucial functions in regulating leaf development, growth, and stress responses in *A. marina*.

### 3.6 Functional analysis of non‐tissue‐specific lncRNAs

**FIGURE 7:**
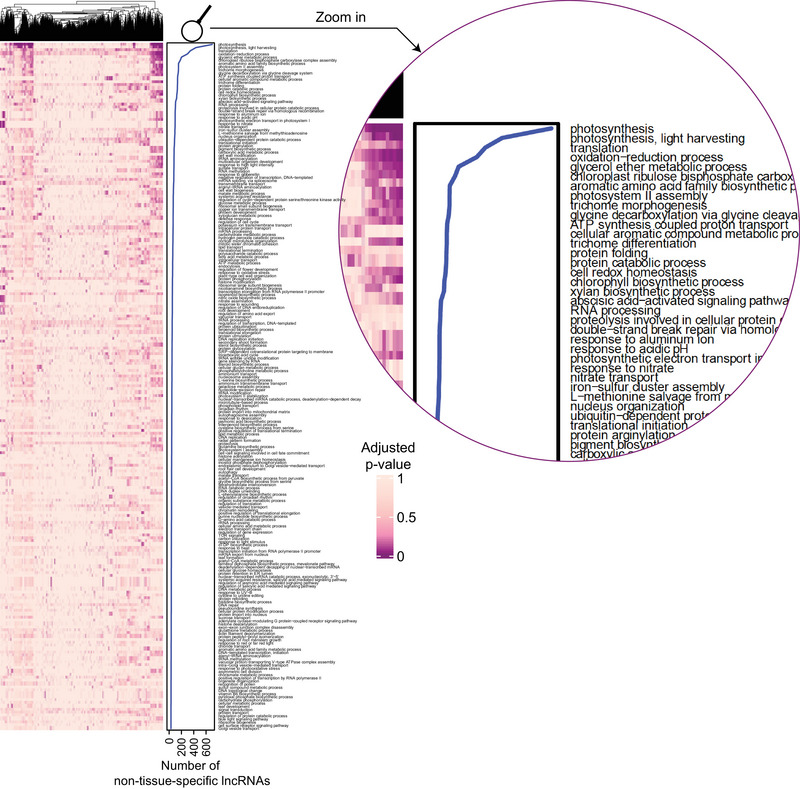
Functional profile of the non‐tissue‐specific long non‐coding RNAs (lncRNAs) in *Avicennia marina*. The biological processes linked to at least 30 non‐tissue‐specific lncRNAs are used for visualization.

Non‐tissue‐specific lncRNAs also constitute an important component of the *A. marina* lncRNAs. Except for the tissue‐specific lncRNAs, we concurrently conducted functional analysis for the non‐tissue‐specific lncRNAs. Our findings revealed that the majority of non‐tissue‐specific lncRNAs are referred to biological processes, such as photosynthesis, translation, glycerol ether metabolic process, chloroplast ribulose bisphosphate carboxylase complex assembly, oxidation–reduction process, and response to aluminum ion, acidic pH, as well as nitrate (Figure 7). This suggests that non‐tissue‐specific lncRNAs are likely to play significant roles in different biological processes involved in both plant growth and environment adaptions across various tissues in *A. marina*.

## 4 DISCUSSION

LncRNAs act as a vital player in diverse biological processes, such as tissue/ organ development, stress responses, aging, and diseases (Statello et al., 2021; Wang et al., 2023; Zhou et al., 2020). Due to the well‐developed high‐throughput sequencing and bioinformatics analysis techniques, thousands of lncRNAs have been identified in various species. Nevertheless, the identification and functional analysis of lncRNAs in *A. marina* have not been conducted yet.

In this study, we integrated the transcriptomic data from multiple tissues of *A. marina* and identified 6333 lncRNAs, which were unevenly distributed on different chromosomes. But for chromosomes 1, 2 and 3, both lncRNAs and mRNAs exhibited a preferred localization on these particular chromosomes. Among these lncRNAs, more than 4000 of them were intergenic lncRNAs, which is consistent with previous research (Wang, Zhao, et al., 2018). The chromosomal distribution of lncRNAs usually showed close relationships with coding genes. These results suggesting that there were more coding genes on chromosome 1, 2 and 3, thus more lncRNAs were found on them. This potentially indicated the importance of these chromosomes during the evolution, growth, and development of *A. marina*.

Besides, these identified lncRNAs presented remarkable higher tissue specificities than mRNAs, which maintain highly consistent with previous research in other plants (Wang et al., 2022). Therefore, hundreds of tissue‐specific lncRNAs were identified from the examined tissues. The tissue‐specific lncRNAs were likely to play important roles in the development and growth of related tissues. These findings suggesting that lncRNAs exhibited more tissue specific than mRNAs, potentially indicating their significant functions in regulating tissues’ development and maintaining their special biological functions (Ma et al., 2021; Zhang et al., 2014). Simultaneously, we found that flower organ‐specific lncRNAs showed an enrichment in cell wall modification and organization processes. Cell wall modification plays a crucial role in the formation of floral organs (Cruz‐Valderrama et al., 2021), indicating that flower‐specific lncRNAs may play a regulatory role in floral organ development.

Additionally, as the underground part of plant, root can hold the plant as well as absorb water and water‐soluble minerals. Meanwhile, root can synthesize organic products, such as amino acids, organic nitrogen, and hormones, which are essential for physiological processes of plant. Besides, several *A. marina* root tissue‐specific lncRNAs were enriched in nitric oxide biosynthetic process, implying their significant functions in root development and stress responses (Ageeva‐Kieferle et al., 2021). Furthermore, we found several leaf‐specific lncRNAs significantly enriched in leaf interrelated biological processes. Especially for *Amarina.29041.3* and *Amarina.1520.3*, as the leaf tissue‐specific lncRNAs, they were significantly enriched in leaf function related processes, such as photosynthesis, photosynthesis light harvesting, and oxidation–reduction process. Interestingly, *Amarina.1520.3* is the potential orthologous lncRNA of CNT2072811 (CANTATAdb ID), which is defined as a confident lncRNA and enriched in response to abiotic and biotic stimulus, response to light stimulus and photosynthesis processes in *Populus trichocarpa* (Szcześniak et al., 2019). In summary, our comprehensive identification, characterization, and functional analysis of the lncRNAs provide theoretical foundation for further studies of lncRNAs in *A. marina* as well as in other plant species.

## AUTHOR CONTRIBUTIONS

**Lingling Wang**: Funding acquisition; project administration; writing—original draft; writing—review and editing. **Zixin Yuan**: Data curation; resources. **Jingyi Wang**: Formal analysis; validation. **Yali Guan**: Investigation; project administration; supervision.

## CONFLICT OF INTEREST STATEMENT

The authors declare no conflicts of interest.

## Supporting information

Figure S1. The density for lncRNAs and mRNAs and classification of the lncRNAs. **(A)** Number of mRNAs across the chromosomes. Density of **(B)** lncRNAs and **(C)** mRNA across the chromosomes. The chromosome length was scaled to 10^6. **(D)** Number of lncRNAs in the different categories.

Figure S2. Tissue specificity of lncRNAs. **(A)** Number of tissue‐specific lncRNAs across the examined tissues. **(B)** Relative abundance of the lncRNAs across the tested tissues. Color density reflects the proportion of lncRNA expression in each tissue in relation to the total expression across all examined tissues.

Figure S3. Three leaf‐specific lncRNAs are associated with a multitude of biological processes. Pink nodes indicate the lncRNAs, while the green nodes represent the biological processes.

Table S1. Sample information of RNA‐seq for this study.

Table S2. Functional enrichment for the flower‐specific lncRNAs.

Table S3. Functional enrichment for the root‐specific lncRNAs.

Table S4. Functional enrichment for the seed‐specific lncRNAs.

Table S5. Functional enrichment for the stem‐specific lncRNAs.

Table S6. Functional enrichment for the leaf‐specific lncRNAs.

Table S7. The co‐expression mRNAs of the lncRNAs *Amarina.29041.3* and *Amarina.1520.3*.

## Data Availability

The data are available in the Supporting Information.

## References

[tpg220450-bib-0001] Ageeva‐Kieferle, A. , Georgii, E. , Winkler, B. , Ghirardo, A. , Albert, A. , Hüther, P. , Mengel, A. , Becker, C. , Schnitzler, J.‐P. , Durner, J. , & Lindermayr, C. (2021). Nitric oxide coordinates growth, development, and stress response via histone modification and gene expression. Plant Physiology, 187, 336–360. 10.1093/plphys/kiab222 34003928 PMC8418403

[tpg220450-bib-0002] Anastasiadou, E. , Jacob, L. S. , & Slack, F. J. (2018). Non‐coding RNA networks in cancer. Nature Reviews Cancer, 18, 5–18. 10.1038/nrc.2017.99 29170536 PMC6337726

[tpg220450-bib-0003] Andrews, S. (2010). FastQC: A quality control tool for high throughput sequence data . http://wwwbioinformaticsbabrahamacuk/projects/fastqc

[tpg220450-bib-0004] Ariel, F. , Romero‐Barrios, N. , Jégu, T. , Benhamed, M. , & Crespi, M. (2015). Battles and hijacks: Noncoding transcription in plants. Trends in Plant Science, 20, 362–371. 10.1016/j.tplants.2015.03.003 25850611

[tpg220450-bib-0005] Batista, P. J. , & Chang, H. Y. (2013). Long noncoding RNAs: Cellular address codes in development and disease. Cell, 152, 1298–1307. 10.1016/j.cell.2013.02.012 23498938 PMC3651923

[tpg220450-bib-0006] Benjamini, Y. , & Hochberg, Y. (1995). Controlling the false discovery rate—A practical and powerful approach to multiple testing. Journal of the Royal Statistical Society: Series B (Methodological), 57, 289–300.

[tpg220450-bib-0007] Bond, A. M. , Vangompel, M. J. W. , Sametsky, E. A. , Clark, M. F. , Savage, J. C. , Disterhoft, J. F. , & Kohtz, J. D. (2009). Balanced gene regulation by an embryonic brain ncRNA is critical for adult hippocampal GABA circuitry. Nature Neuroscience, 12, 1020–1027. 10.1038/nn.2371 19620975 PMC3203213

[tpg220450-bib-0008] Camacho, C. , Coulouris, G. , Avagyan, V. , Ma, N. , Papadopoulos, J. , Bealer, K. , & Madden, T. L. (2009). BLAST plus : Architecture and applications. BMC Bioinformatics, 10, 421. 10.1186/1471-2105-10-421 20003500 PMC2803857

[tpg220450-bib-0009] Charles Richard, J. L. , & Eichhorn, P. J. A. (2018). Platforms for investigating LncRNA functions. SLAS Technology, 23, 493–506. 10.1177/2472630318780639 29945466 PMC6249642

[tpg220450-bib-0010] Chekanova, J. A. , Gregory, B. D. , Reverdatto, S. V. , Chen, H. , Kumar, R. , Hooker, T. , Yazaki, J. , Li, P. , Skiba, N. , Peng, Q. , Alonso, J. , Brukhin, V. , Grossniklaus, U. , Ecker, J. R. , & Belostotsky, D. A. (2007). Genome‐wide high‐resolution mapping of exosome substrates reveals hidden features in the *Arabidopsis* transcriptome. Cell, 131, 1340–1353. 10.1016/j.cell.2007.10.056 18160042

[tpg220450-bib-0011] Cruz‐Valderrama, J. E. , Bernal‐Gallardo, J. J. , Herrera‐Ubaldo, H. , & De Folter, S. (2021). Building a flower: The influence of cell wall composition on flower development and reproduction. Genes, 12(7), 978. 10.3390/genes12070978 34206830 PMC8304806

[tpg220450-bib-0012] Derrien, T. , Johnson, R. , Bussotti, G. , Tanzer, A. , Djebali, S. , Tilgner, H. , Guernec, G. , Martin, D. , Merkel, A. , Knowles, D. G. , Lagarde, J. , Veeravalli, L. , Ruan, X. , Ruan, Y. , Lassmann, T. , Carninci, P. , Brown, J. B. , Lipovich, L. , Gonzalez, J. M. , … Guigó, R. (2012). The GENCODE v7 catalog of human long noncoding RNAs: Analysis of their gene structure, evolution, and expression. Genome Research, 22, 1775–1789. 10.1101/gr.132159.111 22955988 PMC3431493

[tpg220450-bib-0013] Ding, C. , Lim, Y. C. , Chia, S. Y. , Walet, A. C. E. , Xu, S. , Lo, K. A. , Zhao, Y. , Zhu, D. , Shan, Z. , Chen, Q. , Leow, M. K.‐S. , Xu, D. , & Sun, L. (2018). De novo reconstruction of human adipose transcriptome reveals conserved lncRNAs as regulators of brown adipogenesis. Nature communications, 9, 1329. 10.1038/s41467-018-03754-3 PMC5889397 29626186

[tpg220450-bib-0014] Fatica, A. , & Bozzoni, I. (2014). Long non‐coding RNAs: New players in cell differentiation and development. Nature Reviews Genetics, 15, 7–21. 10.1038/nrg3606 24296535

[tpg220450-bib-0015] Haerty, W. , & Ponting, C. P. (2015). Unexpected selection to retain high GC content and splicing enhancers within exons of multiexonic lncRNA loci. RNA, 21, 333–346. 10.1261/rna.047324.114 PMC4338330 25589248

[tpg220450-bib-0016] Han, S. , Liang, Y. , Ma, Q. , Xu, Y. , Zhang, Y. U. , Du, W. , Wang, C. , & Li, Y. (2019). LncFinder: An integrated platform for long non‐coding RNA identification utilizing sequence intrinsic composition, structural information and physicochemical property. Brief Bioinform, 20, 2009–2027. 10.1093/bib/bby065 30084867 PMC6954391

[tpg220450-bib-0017] Huang, X. , Zhang, H. , Wang, Q. , Guo, R. , Wei, L. , Song, H. , Kuang, W. , Liao, J. , Huang, Y. , & Wang, Z. (2021). Genome‐wide identification and characterization of long non‐coding RNAs involved in flag leaf senescence of rice. Plant Molecular Biology, 105, 655–684. 10.1007/s11103-021-01121-3 33569692 PMC7985109

[tpg220450-bib-0018] Jin, J. , Liu, J. , Wang, H. , Wong, L. , & Chua, N.‐H. (2013). PLncDB: Plant long non‐coding RNA database. Bioinformatics, 29, 1068–1071. 10.1093/bioinformatics/btt107 23476021 PMC3624813

[tpg220450-bib-0019] Jones, P. , Binns, D. , Chang, H.‐Y. , Fraser, M. , Li, W. , Mcanulla, C. , Mcwilliam, H. , Maslen, J. , Mitchell, A. , Nuka, G. , Pesseat, S. , Quinn, A. F. , Sangrador‐Vegas, A. , Scheremetjew, M. , Yong, S.‐Y. , Lopez, R. , & Hunter, S. (2014). InterProScan 5: Genome‐scale protein function classification. Bioinformatics, 30, 1236–1240. 10.1093/bioinformatics/btu031 24451626 PMC3998142

[tpg220450-bib-0020] Kang, Y.‐J. , Yang, D.‐C. , Kong, L. , Hou, M. , Meng, Y.‐Q. , Wei, L. , & Gao, G. (2017). CPC2: A fast and accurate coding potential calculator based on sequence intrinsic features. Nucleic Acids Research, 45, W12–W16. 10.1093/nar/gkx428 28521017 PMC5793834

[tpg220450-bib-0021] Kapranov, P. , Cheng, J. , Dike, S. , Nix, D. A. , Duttagupta, R. , Willingham, A. T. , Stadler, P. F. , Hertel, J. , HackermüLler, J. , Hofacker, I. L. , Bell, I. , Cheung, E. , Drenkow, J. , Dumais, E. , Patel, S. , Helt, G. , Ganesh, M. , Ghosh, S. , Piccolboni, A. , … Gingeras, T. R. (2007). RNA maps reveal new RNA classes and a possible function for pervasive transcription. Science, 316, 1484–1488. 10.1126/science.1138341 17510325

[tpg220450-bib-0022] Kavitha, K. , Venkataraman, G. , & Parida, A. (2008). An oxidative and salinity stress induced peroxisomal ascorbate peroxidase from *Avicennia marina*: Molecular and functional characterization. Plant Physiology and Biochemistry, 46, 794–804. 10.1016/j.plaphy.2008.05.008 18614374

[tpg220450-bib-0023] Kim, D. , Paggi, J. M. , Park, C. , Bennett, C. , & Salzberg, S. L. (2019). Graph‐based genome alignment and genotyping with HISAT2 and HISAT‐genotype. Nature Biotechnology, 37, 907–915. 10.1038/s41587-019-0201-4 PMC7605509 31375807

[tpg220450-bib-0024] Kolberg, L. , Raudvere, U. , Kuzmin, I. , Vilo, J. , & Peterson, H. (2020). gprofiler2—An R package for gene list functional enrichment analysis and namespace conversion toolset g:Profiler. F1000Research, 9, ELIXIR709. 10.12688/f1000research.24956.2 PMC7859841 33564394

[tpg220450-bib-0025] Kovaka, S. , Zimin, A. V. , Pertea, G. M. , Razaghi, R. , Salzberg, S. L. , & Pertea, M. (2019). Transcriptome assembly from long‐read RNA‐seq alignments with StringTie2. Genome Biology, 20, 278. 10.1186/s13059-019-1910-1 31842956 PMC6912988

[tpg220450-bib-0026] Kuenzer, C. , Bluemel, A. , Gebhardt, S. , Quoc, T. V. , & Dech, S. (2011). Remote sensing of mangrove ecosystems: A review. Remote Sensing, 3, 878–928. 10.3390/rs3050878

[tpg220450-bib-0027] Lawrence, M. , Huber, W. , Pagès, H. , Aboyoun, P. , Carlson, M. , Gentleman, R. , Morgan, M. T. , & Carey, V. J. (2013). Software for computing and annotating genomic ranges. Plos Computational Biology, 9, e1003118. 10.1371/journal.pcbi.1003118 23950696 PMC3738458

[tpg220450-bib-0028] Li, H.‐L. , Wang, Y. , Guo, D. , Zhu, J.‐H. , & Peng, S.‐Q. (2021). Differential expression of lncRNAs and miRNAs between self‐rooting juvenile and donor clones unveils novel insight into the molecular regulation of rubber biosynthesis in *Hevea brasiliensis* . Frontiers in Plant Science, 12, 740597. 10.3389/fpls.2021.740597 35069613 PMC8767119

[tpg220450-bib-0029] Li, L. , Eichten, S. R. , Shimizu, R. , Petsch, K. , Yeh, C. T. , Wu, W. , Chettoor, A. M. , Givan, S. A. , Cole, R. A. , Fowler, J. E. , Evans, M. M. S. , Scanlon, M. J. , Yu, J. , Schnable, P. S. , Timmermans, M. C. P. , Springer, N. M. , & Muehlbauer, G. J. (2014). Genome‐wide discovery and characterization of maize long non‐coding RNAs. Genome Biology, 15, R40. 10.1186/gb-2014-15-2-r40 24576388 PMC4053991

[tpg220450-bib-0030] Lin, X. , Lin, W. , Ku, Y.‐S. , Wong, F.‐L. , Li, M.‐W. , Lam, H.‐M. , Ngai, S.‐M. , & Chan, T.‐F. (2020). Analysis of soybean long non‐coding RNAs reveals a subset of small peptide‐coding transcripts. Plant Physiology, 182, 1359–1374. 10.1104/pp.19.01324 31882456 PMC7054870

[tpg220450-bib-0031] Liu, F. , Xu, Y. , Chang, K. , Li, S. , Liu, Z. , Qi, S. , Jia, J. , Zhang, M. , Crawford, N. M. , & Wang, Y. (2019). The long noncoding RNA T5120 regulates nitrate response and assimilation in *Arabidopsis* . New Phytologist, 224, 117–131. 10.1111/nph.16038 31264223

[tpg220450-bib-0032] Liu, H. , Hu, Y. , Yuan, K. , Feng, C. , He, Q. , Sun, L. , & Wang, Z. (2022). Genome‐wide identification of lncRNAs, miRNAs, mRNAs and their regulatory networks involved in tapping panel dryness in rubber tree (*Hevea brasiliensis*). Tree Physiology, 42, 629–645. 10.1093/treephys/tpab120 34533196

[tpg220450-bib-0033] Ma, B. , Zhang, A. , Zhao, Q. , Li, Z. , Lamboro, A. , He, H. , Li, Y. , Jiao, S. , Guan, S. , Liu, S. , Yao, D. , & Zhang, J. (2021). Genome‐wide identification and analysis of long non‐coding RNAs involved in fatty acid biosynthesis in young soybean pods. Scientific Reports, 11, 7603. 10.1038/s41598-021-87048-7 33828134 PMC8027399

[tpg220450-bib-0034] Mistry, J. , Chuguransky, S. , Williams, L. , Qureshi, M. , Salazar, G. A. , Sonnhammer, E. L. L. , Tosatto, S. C. E. , Paladin, L. , Raj, S. , Richardson, L. J. , Finn, R. D. , & Bateman, A. (2021). Pfam: The protein families database in 2021. Nucleic Acids Research, 49, D412–D419. 10.1093/nar/gkaa913 33125078 PMC7779014

[tpg220450-bib-0035] Moison, M. , Pacheco, J. M. , Lucero, L. , Fonouni‐Farde, C. , Rodríguez‐Melo, J. , Mansilla, N. , Christ, A. , Bazin, J. , Benhamed, M. , Ibañez, F. , Crespi, M. , Estevez, J. M. , & Ariel, F. (2021). The lncRNA APOLO interacts with the transcription factor WRKY42 to trigger root hair cell expansion in response to cold. Molecular Plant, 14, 937–948. 10.1016/j.molp.2021.03.008 33689931

[tpg220450-bib-0036] Natarajan, P. , Murugesan, A. K. , Govindan, G. , Gopalakrishnan, A. , Kumar, R. , Duraisamy, P. , Balaji, R. , Tanuja , Shyamli, P. S. , Parida, A. K. , & Parani, M. (2021). A reference‐grade genome identifies salt‐tolerance genes from the salt‐secreting mangrove species *Avicennia marina* . Communications Biology, 4, 851. 10.1038/s42003-021-02384-8 34239036 PMC8266904

[tpg220450-bib-0037] Negri, T. D. C. , Alves, W. A. L. , Bugatti, P. H. , Saito, P. T. M. , Domingues, D. S. , & Paschoal, A. R. (2019). Pattern recognition analysis on long noncoding RNAs: A tool for prediction in plants. Briefings in Bioinformatics, 20, 682–689. 10.1093/bib/bby034 29697740

[tpg220450-bib-0038] Pang, J. , Zhang, X. , Ma, X. , & Zhao, J. (2019). Spatio‐temporal transcriptional dynamics of maize long non‐coding RNAs responsive to drought stress. Genes, 10(2), 138. 10.3390/genes10020138 30781862 PMC6410058

[tpg220450-bib-0039] Patro, R. , Duggal, G. , Love, M. I. , Irizarry, R. A. , & Kingsford, C. (2017). Salmon provides fast and bias‐aware quantification of transcript expression. Nature Methods, 14, 417–419. 10.1038/nmeth.4197 28263959 PMC5600148

[tpg220450-bib-0040] Qin, T. , Zhao, H. , Cui, P. , Albesher, N. , & Xiong, L. (2017). A nucleus‐localized long non‐coding RNA enhances drought and salt stress tolerance. Plant Physiology, 175, 1321–1336. 10.1104/pp.17.00574 28887353 PMC5664461

[tpg220450-bib-0041] Quinn, J. J. , & Chang, H. Y. (2016). Unique features of long non‐coding RNA biogenesis and function. Nature Reviews Genetics, 17, 47–62. 10.1038/nrg.2015.10 26666209

[tpg220450-bib-0042] Robinson, M. D. , Mccarthy, D. J. , & Smyth, G. K. (2010). edgeR: A Bioconductor package for differential expression analysis of digital gene expression data. Bioinformatics, 26, 139–140. 10.1093/bioinformatics/btp616 19910308 PMC2796818

[tpg220450-bib-0043] Schubert, M. , Lindgreen, S. , & Orlando, L. (2016). AdapterRemoval v2: Rapid adapter trimming, identification, and read merging. BMC Research Notes, 9, Article 88. 10.1186/s13104-016-1900-2 26868221 PMC4751634

[tpg220450-bib-0044] Statello, L. , Guo, C.‐J. , Chen, L.‐L. , & Huarte, M. (2021). Gene regulation by long non‐coding RNAs and its biological functions. Nature Reviews Molecular Cell Biology, 22, 96–118. 10.1038/s41580-020-00315-9 33353982 PMC7754182

[tpg220450-bib-0045] Szcześniak, M. W. , Bryzghalov, O. , Ciomborowska‐Basheer, J. , & Makałowska, I. (2019). CANTATAdb 2.0: Expanding the collection of plant long noncoding RNAs. Plant Long Non‐Coding RNAs, 1933, 415–429. 10.1007/978-1-4939-9045-0_26 30945201

[tpg220450-bib-0046] Tian, R. , Sun, X. , Liu, C. , Chu, J. , Zhao, M. , & Zhang, W.‐H. (2023). A medicago truncatula lncRNA MtCIR1 negatively regulates response to salt stress. Planta, 257(2), Article 32. 10.1007/s00425-022-04064-1 36602592

[tpg220450-bib-0047] Tian, Y. , Zheng, H. , Zhang, F. , Wang, S. , Ji, X. , Xu, C. , He, Y. , & Ding, Y. (2019). PRC2 recruitment and H3K27me3 deposition at FLC require FCA binding of COOLAIR. Science Advances, 5, eaau7246. 10.1126/sciadv.aau7246 31032401 PMC6482009

[tpg220450-bib-0048] Wan, S. , Zhang, Y. , Duan, M. , Huang, L. , Wang, W. , Xu, Q. , Yang, Y. , & Yu, Y. (2020). Integrated analysis of long non‐coding RNAs (lncRNAs) and mRNAs reveals the regulatory role of lncRNAs associated with salt resistance in *Camellia sinensis* . Frontiers in Plant Science, 11, Article 218. 10.3389/fpls.2020.00218 32265948 PMC7096555

[tpg220450-bib-0049] Wang, H. , Chung, P. J. , Liu, J. , Jang, I.‐C. , Kean, M. J. , Xu, J. , & Chua, N.‐H. (2014). Genome‐wide identification of long noncoding natural antisense transcripts and their responses to light in *Arabidopsis* . Genome Research, 24, 444–453. 10.1101/gr.165555.113 24402519 PMC3941109

[tpg220450-bib-0050] Wang, L. , Wang, J. , Chen, H. , & Hu, B. (2022). Genome‐wide identification, characterization, and functional analysis of lncRNAs in Hevea brasiliensis. Frontiers in Plant Science, 13, 1012576. 10.3389/fpls.2022.1012576 36275565 PMC9581277

[tpg220450-bib-0051] Wang, M. , Zhao, W. , Gao, L. , & Zhao, L. (2018). Genome‐wide profiling of long non‐coding RNAs from tomato and a comparison with mRNAs associated with the regulation of fruit ripening. BMC Plant Biology, 18, 75. 10.1186/s12870-018-1300-y 29728060 PMC5935960

[tpg220450-bib-0052] Wang, X. , Fan, H. , Wang, B. , & Yuan, F. (2023). Research progress on the roles of lncRNAs in plant development and stress responses. Frontiers in Plant Science, 14, 1138901. 10.3389/fpls.2023.1138901 36959944 PMC10028117

[tpg220450-bib-0053] Wang, Y. , Fan, X. , Lin, F. , He, G. , Terzaghi, W. , Zhu, D. , & Deng, X. W. (2014). *Arabidopsis* noncoding RNA mediates control of photomorphogenesis by red light. PNAS, 111, 10359–10364. 10.1073/pnas.1409457111 24982146 PMC4104870

[tpg220450-bib-0054] Wang, Y. , Li, J. , Deng, X.‐W. , & Zhu, D. (2018). *Arabidopsis* noncoding RNA modulates seedling greening during deetiolation. Science China Life Sciences, 61, 199–203. 10.1007/s11427-017-9187-9 29143279

[tpg220450-bib-0055] Wang, Y. , Luo, X. , Sun, F. , Hu, J. , Zha, X. , Su, W. , & Yang, J. (2018). Overexpressing lncRNA LAIR increases grain yield and regulates neighbouring gene cluster expression in rice. Nature Communications, 9, 3516. 10.1038/s41467-018-05829-7 PMC6115402 30158538

[tpg220450-bib-0056] Wierzbicki, A. T. (2012). The role of long non‐coding RNA in transcriptional gene silencing. Current Opinion in Plant Biology, 15, 517–522. 10.1016/j.pbi.2012.08.008 22960034

[tpg220450-bib-0057] Wilusz, J. E. , Sunwoo, H. , & Spector, D. L. (2009). Long noncoding RNAs: Functional surprises from the RNA world. Genes & Development, 23, 1494–1504. 10.1101/gad.1800909 19571179 PMC3152381

[tpg220450-bib-0058] Wucher, V. , Legeai, F. , Hédan, B. , Rizk, G. , Lagoutte, L. , Leeb, T. , Jagannathan, V. , Cadieu, E. , David, A. , Lohi, H. , Cirera, S. , Fredholm, M. , Botherel, N. , Leegwater, P. A. J. , Le Béguec, C. , Fieten, H. , Johnson, J. , Alföldi, J. , André, C. , … Derrien, T. (2017). FEELnc: A tool for long non‐coding RNA annotation and its application to the dog transcriptome. Nucleic Acids Research, 45, e57. 10.1093/nar/gkw1306 28053114 PMC5416892

[tpg220450-bib-0059] Yin, H. , Zhang, X. , Zhang, B. , Luo, H. , & He, C. (2019). Revealing the dominant long noncoding RNAs responding to the infection with *Colletotrichum gloeosporioides* in *Hevea brasiliensis* . Biology Direct, 14, Article 7. 10.1186/s13062-019-0235-z 30987641 PMC6466799

[tpg220450-bib-0060] Yu, Y. , Zhang, Y. , Chen, X. , & Chen, Y. (2019). Plant noncoding RNAs: Hidden players in development and stress responses. Annual Review of Cell and Developmental Biology, 35, 407–431. 10.1146/annurev-cellbio-100818-125218 PMC8034839 31403819

[tpg220450-bib-0061] Zeinali, F. , Homaei, A. , & Kamrani, E. (2017). Identification and kinetic characterization of a novel superoxide dismutase from Avicennia marina: An antioxidant enzyme with unique features. International Journal of Biological Macromolecules, 105, 1556–1562. 10.1016/j.ijbiomac.2017.07.054 28720545

[tpg220450-bib-0062] Zhang, Y.‐C. , Liao, J.‐Y. , Li, Z.‐Y. , Yu, Y. , Zhang, J.‐P. , Li, Q.‐F. , Qu, L.‐H. , Shu, W.‐S. , & Chen, Y.‐Q. (2014). Genome‐wide screening and functional analysis identify a large number of long noncoding RNAs involved in the sexual reproduction of rice. Genome Biology, 15, 512. 10.1186/s13059-014-0512-1 25517485 PMC4253996

[tpg220450-bib-0063] Zhou, Q. , Wan, Q. , Jiang, Y. , Liu, J. , Qiang, L. I. , & Sun, L. (2020). A landscape of murine long non‐coding RNAs reveals the leading transcriptome alterations in adipose tissue during aging. Cell Reports, 31, 107694. 10.1016/j.celrep.2020.107694 32460027 PMC7603645

