# Supplementary material for: Genome‐wide identification and functional profile analysis of long non‐coding RNAs in Avicennia marina
Source: Plant Genome. 2024 Apr 11;17(2):e20450. doi: 10.1002/tpg2.20450 (PMC12807275; doi:10.1002/tpg2.20450)
Supplement: Supplementary file 1 [file TPG2-17-e20450-s001.pdf]

Figure S3

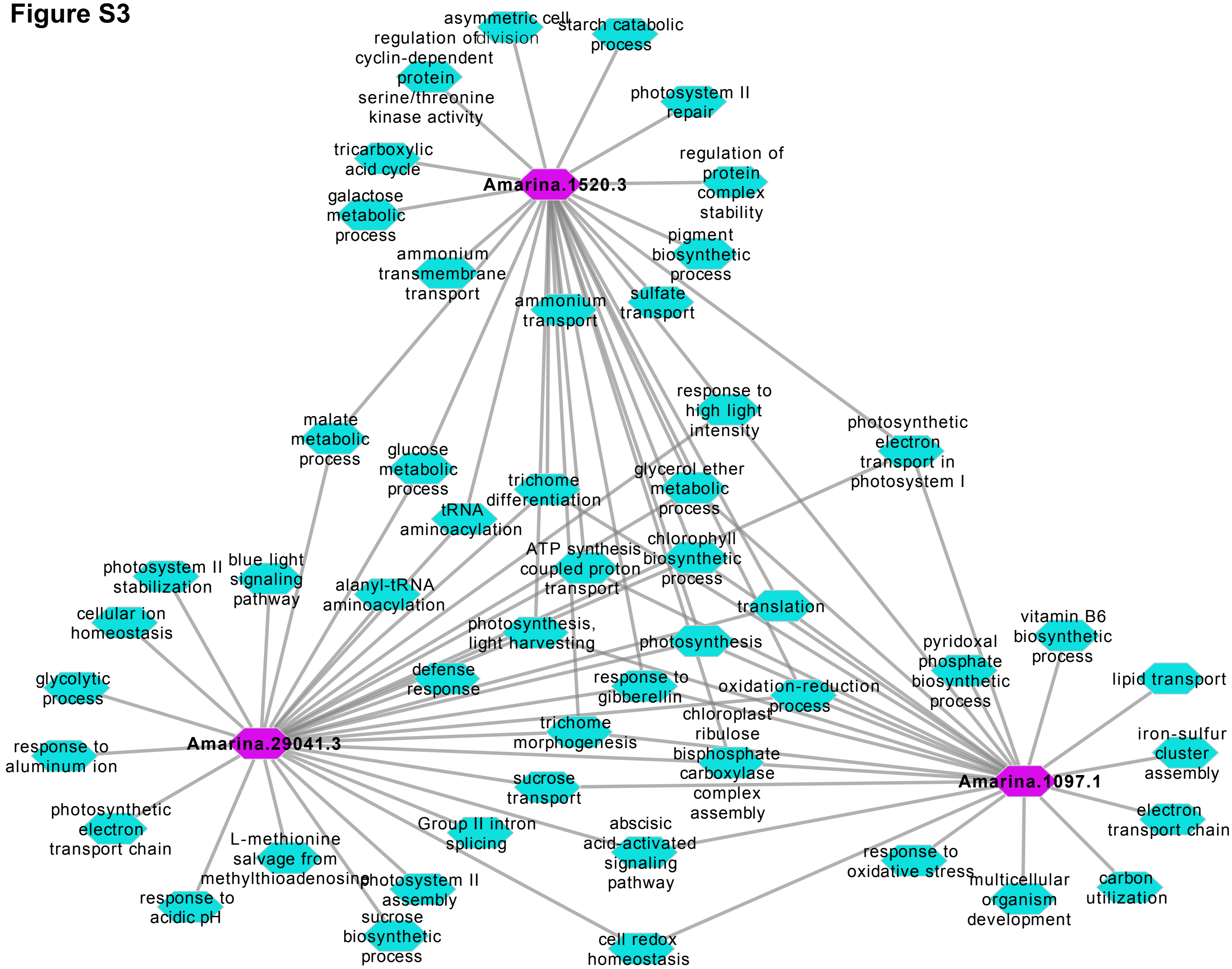
asymmetric cell division
starch catabolic process
regulation of cyclin-dependent protein serine/threonine kinase activity
photosystem II repair
tricarboxylic acid cycle
Amarina.1520.3
regulation of protein complex stability
galactose metabolic process
pigment biosynthetic process
ammonium transmembrane transport
ammonium transport
sulfate transport
response to high light intensity
photosynthetic electron transport in photosystem I
malate metabolic process
glucose metabolic process
trichome differentiation
glycerol ether metabolic process
tRNA aminoacylation
ATP synthesis coupled proton transport
chlorophyll biosynthetic process
photosystem II stabilization
blue light signaling pathway
alanyl-tRNA aminoacylation
translation
cellular ion homeostasis
photosynthesis, light harvesting
photosynthesis
vitamin B6 biosynthetic process
pyridoxal phosphate biosynthetic process
glycolytic process
defense response
response to gibberellin
oxidation-reduction process
lipid transport
trichome morphogenesis
chloroplast ribulose bisphosphate carboxylase complex assembly
iron-sulfur cluster assembly
response to aluminum ion
Amarina.29041.3
Amarina.1097.1
sucrose transport
photosynthetic electron transport chain
electron transport chain
L-methionine salvage from methylthioadenosine
Group II intron splicing
abscisic acid-activated signaling pathway
response to oxidative stress
multicellular organism development
carbon utilization
photosystem II assembly
response to acidic pH
sucrose biosynthetic process
cell redox homeostasis
