# Supplementary material for: Genome‐wide identification and functional profile analysis of long non‐coding RNAs in Avicennia marina
Source: Plant Genome. 2024 Apr 11;17(2):e20450. doi: 10.1002/tpg2.20450 (PMC12807275; doi:10.1002/tpg2.20450)
Supplement: Supplementary file 2 [file TPG2-17-e20450-s002.pdf]

Figure S1

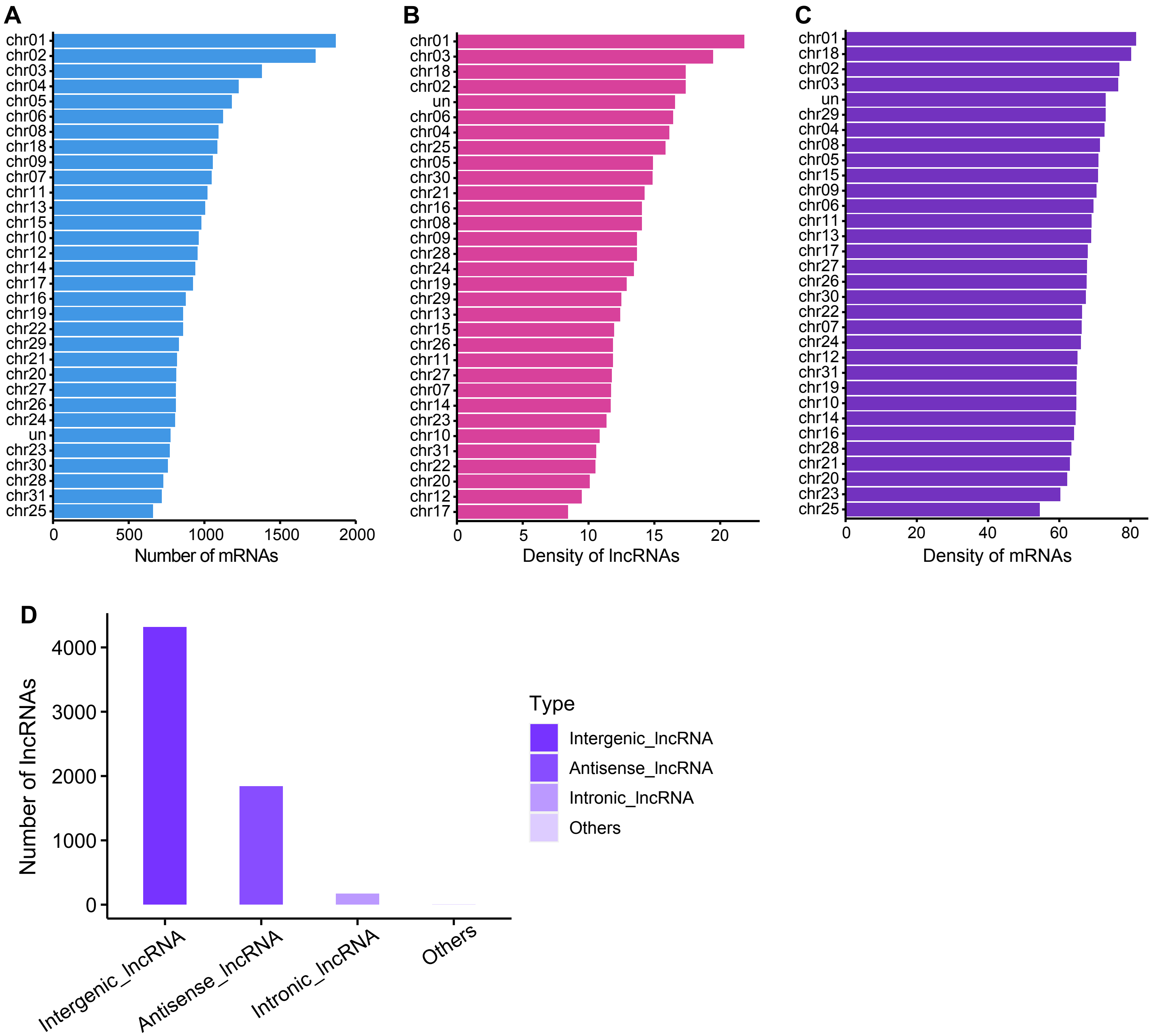
A
chr01
chr02
chr03
chr04
chr05
chr06
chr08
chr18
chr09
chr07
chr11
chr13
chr15
chr10
chr12
chr14
chr17
chr16
chr19
chr22
chr29
chr21
chr20
chr27
chr26
chr24
un
chr23
chr30
chr28
chr31
chr25
0
500
1000
1500
2000
Number of mRNAs
B
chr01
chr03
chr18
chr02
un
chr06
chr04
chr25
chr05
chr30
chr21
chr16
chr08
chr09
chr28
chr24
chr19
chr29
chr13
chr15
chr26
chr11
chr27
chr07
chr14
chr23
chr10
chr31
chr22
chr20
chr12
chr17
0
5
10
15
20
Density of lncRNAs
C
chr01
chr18
chr02
chr03
un
chr29
chr04
chr08
chr05
chr15
chr09
chr06
chr11
chr13
chr17
chr27
chr26
chr30
chr22
chr07
chr24
chr12
chr31
chr19
chr10
chr14
chr16
chr28
chr21
chr20
chr23
chr25
0
20
40
60
80
Density of mRNAs
D
Number of lncRNAs
4000
3000
2000
1000
0
Intergenic_lncRNA
Antisense_lncRNA
Intronic_lncRNA
Others
Type
Intergenic_lncRNA
Antisense_lncRNA
Intronic_lncRNA
Others
