# Supplementary material for: Genome‐wide identification and functional profile analysis of long non‐coding RNAs in Avicennia marina
Source: Plant Genome. 2024 Apr 11;17(2):e20450. doi: 10.1002/tpg2.20450 (PMC12807275; doi:10.1002/tpg2.20450)
Supplement: Supplementary file 3 [file TPG2-17-e20450-s006.pdf]

Figure S2

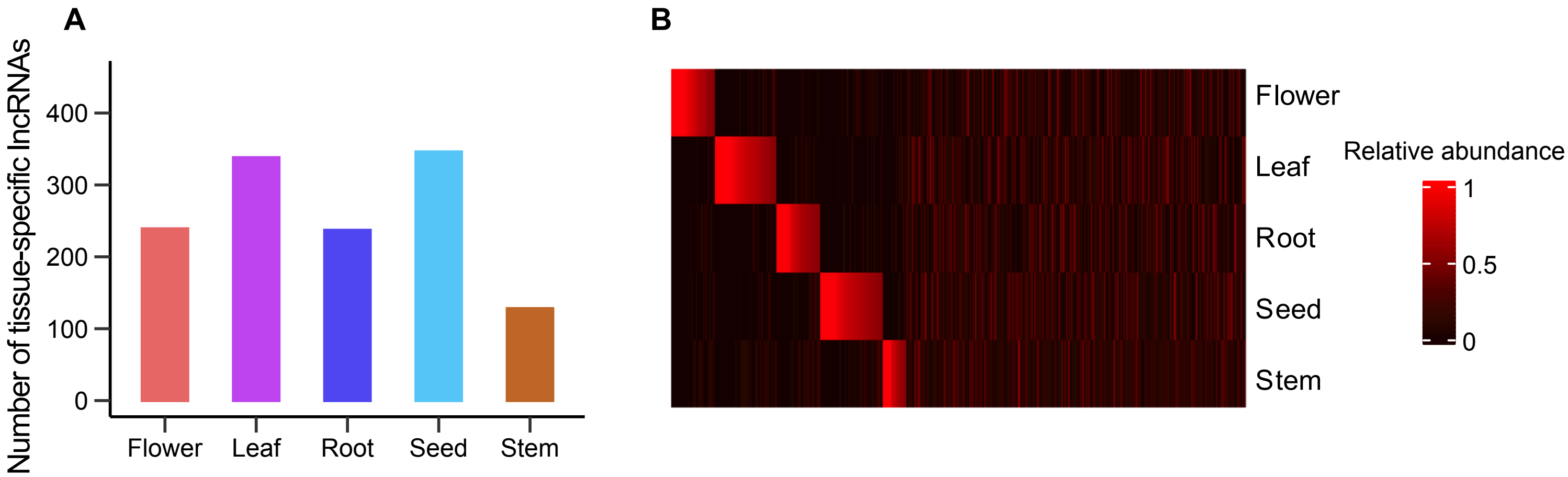
A
Number of tissue-specific lncRNAs
0
100
200
300
400
Flower
Leaf
Root
Seed
Stem
B
Flower
Leaf
Root
Seed
Stem
Relative abundance
1
0.5
0
